# The Computerized Medical Record as a Tool for Clinical Governance in Australian Primary Care

**DOI:** 10.2196/ijmr.2700

**Published:** 2013-08-12

**Authors:** Christopher Martin Pearce, Simon de Lusignan, Christine Phillips, Sally Hall, Joanne Travaglia

**Affiliations:** ^1^Inner East Melbourne Medicare LocalBurwood EastAustralia; ^2^Department of General PracticeMonash UniversityClaytonAustralia; ^3^Department of Healthcare Management and PolicyUniversity of SurreyGuildfordUnited Kingdom; ^4^Academic Unit of General Practice and Community HealthAustralian National UniversityCanberraAustralia; ^5^Centre for Clinical Governance and HealthFaculty of MedicineUniversity of New South WalesSydneyAustralia

**Keywords:** clinical governance, electronic health records, general practice, realist evaluation, quality assurance, health care

## Abstract

**Background:**

Computerized medical records (CMR) are used in most Australian general practices. Although CMRs have the capacity to amalgamate and provide data to the clinician about their standard of care, there is little research on the way in which they may be used to support clinical governance: the process of ensuring quality and accountability that incorporates the obligation that patients are treated according to best evidence.

**Objective:**

The objective of this study was to explore the capability, capacity, and acceptability of CMRs to support clinical governance.

**Methods:**

We conducted a realist review of the role of seven CMR systems in implementing clinical governance, developing a four-level maturity model for the CMR. We took Australian primary care as the context, CMR to be the mechanism, and looked at outcomes for individual patients, localities, and for the population in terms of known evidence-based surrogates or true outcome measures.

**Results:**

The lack of standardization of CMRs makes national and international benchmarking challenging. The use of the CMR was largely at level two of our maturity model, indicating a relatively simple system in which most of the process takes place outside of the CMR, and which has little capacity to support benchmarking, practice comparisons, and population-level activities. Although national standards for coding and projects for record access are proposed, they are not operationalized.

**Conclusions:**

The current CMR systems can support clinical governance activities; however, unless the standardization and data quality issues are addressed, it will not be possible for current systems to work at higher levels.

## Introduction

Clinical governance is an approach to ensuring quality and accountability that incorporates the obligation that patients are treated according to best evidence (Textbox 1).

Computerized medical records (CMR-see Textbox 2) provide a viable mechanism for implementing clinical governance [1]. Computers are involved in all aspects of the clinical interaction-from consulting room to system-level use of large systems that might control entitlement to treatment, screening, recall, and on-line booking of services [2,3]. In Australia, the UK and Netherlands, primary care is highly computerized, with almost all primary care physicians using a CMR; while in the US and Canada, primary care is less computerized, with the hospital sector leading the way [3]. Between 20% and 40% of the clinical consultation is spent interacting with the computer [4-6].

It is important to understand the context within which records are created [7]. Simply having a CMR does not guarantee the creation of a complete record usable for clinical governance purposes; the interaction with the computer in the consultation is complex and evolving [8]. Using a CMR is not a neutral act [9]; there are barriers to using the computer and coding systems [10] and interfacing with them constrains what is recorded [11,12]. However, the CMR does enable the running of decision support programs that can reduce errors, [13] and it can improve quality though audit/feedback cycles [14]. There are issues about the governance of these records and the repositories derived from these data; and formal governance structures are often lacking [15].

We carried out this investigation to see, firstly, how the nature of the design of different vendors’ CMR systems enable and constrain clinical governance and, secondly, how individuals and groups might use computers differently as tools to measure quality and to achieve clinical governance objectives. We describe an assessment tool that would enable others to assess the extent to which any CMR could act as a mechanism within their health care context to support clinical governance.

**What is clinical governance?**

Clinical governance is a term first used within the UK National Health Service (NHS) to describe a process for maintenance, improvement, monitoring, and accountability for clinical standards.

The NHS clinical governance process made chief executives responsible and accountable for clinical quality in their organization alongside business goals and budgetary control.

Clinical governance also challenged the “clinical freedom” of doctors. Up to its inception, a doctor only needed to justify their actions in terms of them judged to be reasonable by a group of peers. The onus changed to one where clinicians are expected to deliver best practice, usually as defined in evidence-based guidelines, and to participate in clinical audits of their standards of care.

Persistent deviation from guidelines, or being an outlier in audit might be the cause for review.

**Terminology used in the relation to computerized medical records.**

Classification system

A range of terms exists to describe CMR systems. The classifications reflect differences in the following areas:

1. Purpose: Intended to be a life-long or partial record of a patient’s health or medical treatment.
2. Disciplinary base: Based on the “medical model” or a broader “health” record.
3. Proprietorship: Owned and controlled by the patient or their proxy, or by the health care provider or health care system.

Definitions:

*Electronic health record (EHR):* A complete “cradle to grave” record of the health and health care provided to an individual.

*Computer-based patient record:* Historic term for an EHR-like concept. Sometimes used to indicate “all health related data”.

*Electronic medical record*: Records of the part of a patients care held by a specific medical provider or department. Health care providers generally aim for these to be enterprise wide.

*Electronic patient record:* Similar to EHR, a lifelong record of health and health care provided.

*Personal health records*: A complete medical and health care record controlled by the patient or their proxy.

Our preferred term:

*Computerized medical record (CMR*): This is a generic term, similar to digital medical record, which implies that this is a record under the custodianship of a medical or health care provider, and is inclusive of partial and complete records.

## Methods

### Background

This study was a component of a larger systematic review and realist synthesis of clinical governance in primary care [16]. The CMR had the allure of being an unrealized tool to support clinical governance, measuring quality, conducting clinical audit, and ensuring safety (Textbox 3). We therefore undertook an analysis of CMR systems used in Australia, exploring the extent to which the CMR supported clinical governance, including to what extent this reflects contextual factors that may be unique to the Australian context. In keeping with the main study framework, we performed a structured analysis in conjunction with key themes emerging in the main study from a literature review and informant interviews. We analyzed seven CMR products used in Australia, and also their capacity to deliver clinical governance. We concluded by developing a maturity framework for CMRs in relation to clinical governance, and classified the maturity of the various CMRs.

**Scope and role of an information system to support clinical governance.**

Computerized information systems can use routine data, and specially captured additional data (eg, patient questionnaires) to audit quality.

Clinical governance makes demands of managers, clinicians, and information systems:

- Managers: Responsible and accountable for clinical standards within their organization; including mechanism for measuring them.
- Doctors: Clinicians are now expected to deliver best practice as defined in evidence-based guidelines; and participate in clinical audit.
- Information systems: It should play a role beyond individual patient care, practice, and locality audits. The CMR should enable practices to benchmark quality, and governments to see there is return on investment by ensuring it supports evidence-based practice.

Patients’ views of the service and their “experience” of healthcare are an important measure of quality, which is missing in current systems.

### Realist Evaluation

We carried out a review from a realist perspective, mirroring the approach of the main study [17], modifying the approach previously used to explore the success and failures of the UK National program for IT [18]. A realist perspective is useful in assessing complex interventions as it aims to develop explanatory analyses of why and how these interventions may work in particular settings and contexts. The realists mantra is: “Context (C)” plus causal link with an appropriate “Mechanism (M)” results in an “Outcome (O)”; in other words, “C+M=O”. Part of the realist perspective is that effects are reported according to the three Ws: “*What Works, for Whom, and in What circumstances*.”

**Figure 1:**
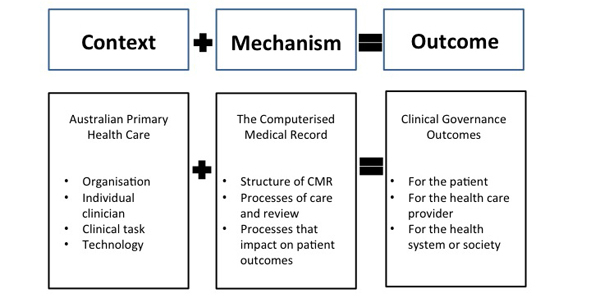
Overview model of the method to appraise whether in the context of Australian primary care the CMR provided a mechanism for driving clinical governance.

In realist evaluations, there can sometimes be difficulty in distinguishing context and mechanism. In this analysis, the context (C) is the Australian primary health care context, and the mechanism (M) is the CMR system used at the point of care. Our outcome measure (O) was the ability to produce clinical governance outputs through the ability to monitor quality of care against given criteria and standards. This combination describes how in the Australian context (C), the CMR, might contribute as a mechanism (M) to deliver the outcome measure (O), clinical governance (C+M=O) (Figure 1).

### Context

We mapped the primary care context using Lusignan’s 4 component classification [8]:

1. Organization: We considered the ways in which primary health care was organized at the practice, locality, and at the national level in Australia.
2. Individual clinicians: We considered the level of knowledge, skill in operation, and attitude toward CMR among individual general practitioners.
3. Clinical task: We considered the clinical context during which the CMR would be used. This was usually a one-to-one clinical consultation, in which the presence of the desk-based CMR created a triadic clinical relationship [19].
4. Technology: We considered the features of the technology, which are unique to the particular context. Australia is in the process of enhancing broadband access, but this is unequally distributed around the country.

The contextual features discovered through this review were then analyzed in concert with the mechanisms of the CMR described below to develop a nuanced understanding of how the CMR operated in this particular environment to produce governance outcomes.

### Mechanism

To identify the ways in which the CMR operated in relation to clinical governance, we used the Donabedian [20] classification of structure and process elements to describe the three types of mechanism by which CMRs may enable the delivery of improved clinical governance: structures, processes of care and review, and processes that impact on outcome. In this study, the software settings were considered to be process elements. For example, a key enabler of clinical governance-such as the presence of a unique patient identifier within the system, essential for data aggregation-would be listed as a key component of the mechanism provided by the CMR.

1. “Structures” included the physical structures and design features (including conventions for room layout, record architecture, and linkages).
2. “Processes of care and review” included software capabilities such as the issuing of prescriptions.
3. “Processes that may impact upon on patient outcomes” included elements such as the ability of the CMR system to detect and block all serious drug interactions.

Each of these categories was subcategorized to produce the detailed tool across the categories (Table 1). The CMR structure was divided using the Open EHR model of the four separate components of a CMR system: interface, clinical archetypes, coding system, and database.

### Outcome

We explored factors related to clinical governance outcomes occurring at the level of the patient, the health care provider, and the setting (ie, impact at the population- or health system-level) [21].

### Assessment Tool

We created a new assessment tool (Multimedia Appendix 1), a bi-axial tool, where the previously-described taxonomies of mechanism and context occupied each of the axes. The cells of the grid are populated with outcomes related to clinical governance for patients/clients, the provider, and the broader population level.

### Assessing the Top Six Brands and One Example of a CMR System With a Low User Base

The top six CMR systems measured in terms of user base [22] were evaluated using this tool (Textbox 4). We also examined a CMR system with a small installation base (and therefore less organizational resources within the company) as a comparator. For each system, we used either software in demonstration mode or installed software in training mode. The testing was done with simulated patient data, and independently of the software providers, to explore how the system might retain clinical data and enable clinical governance activities. The tool was applied by one researcher and checked for accuracy by experienced users of each system. We elected not to disclose or publish comparison between brands, instead keeping our focus on whether the current generation of CMRs provide a viable mechanism for implementing clinical governance.

### Maturity Framework

**Figure 2:**
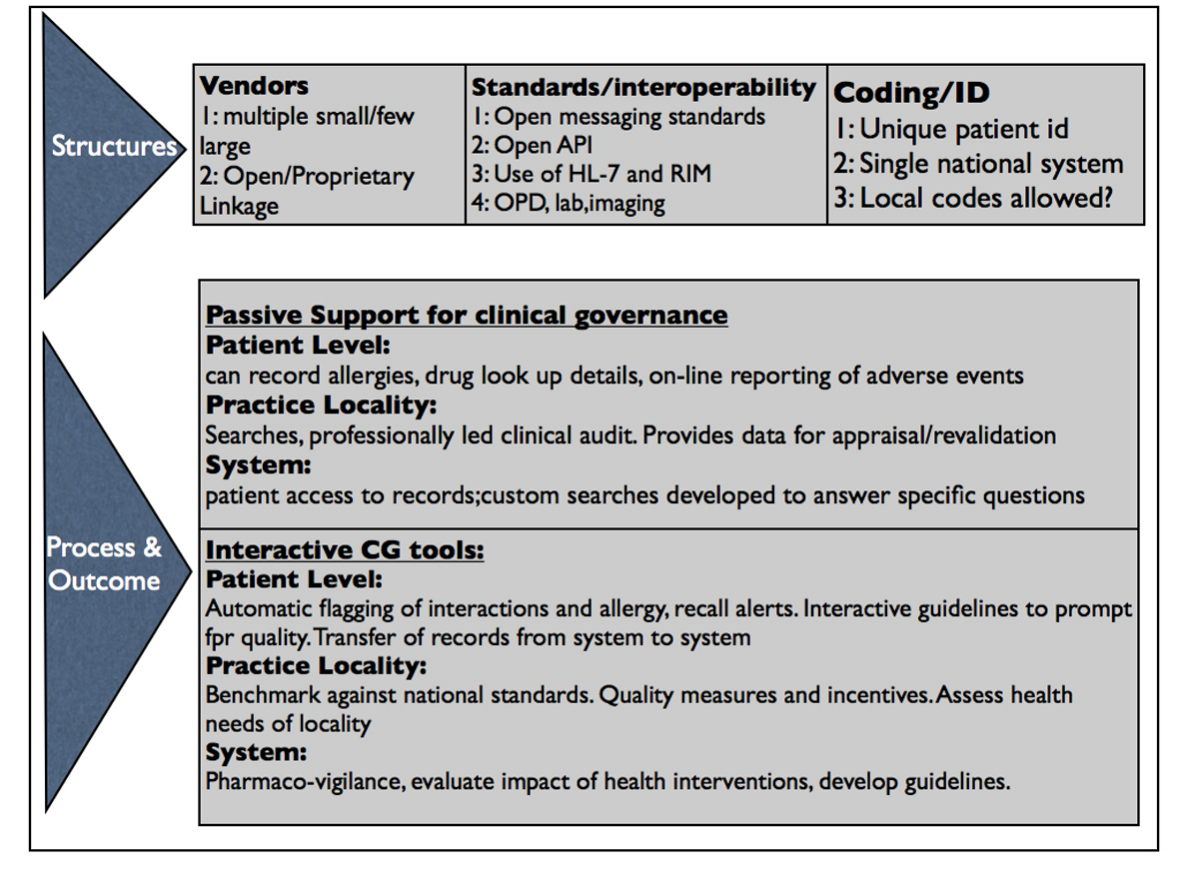
Schema of the maturity framework.

We developed a CMR maturity model, again using the Donabedian classification into structures (including IT architecture issues), process and outcomes, using existing consensus about CMR maturity [23-25]. At the structural level, we looked at the number of vendors and their market share, use of standards and interoperability, and the use of unique patient identifiers and clinical coding (eg, single national coding system). The processes were graded from passive reporting through to active decision support-again looking at individual patient, practice or locality and population levels. Outcomes data were expressed in terms of feedback about quality (Figure 2).

The process and potential of the CMR to influence clinical governance outcomes were graded into a four-level model (Table 2). This grading is multi-dimensional: (1) the agency of the CMR: namely, does the CMR play a passive or dependent role compared to an active or autonomous role in delivering clinical governance, (2) the level of complexity of the transaction and whether or not it is adaptive [26], (3) the degree of integration with other information systems, and (4) the physical integration and linkage processes underpinning it.

**Table 1:** Donabedian based assessment of CMR as a mechanism to support clinical governance.

Structures		Element explored
System Architecture (eg, Open EPR model)		Interface, clinical archetypes, database type, coding systems
Information & Decision Support		Drug databases, interactions, clinical calculators
System Linkages		Patient registrations, laboratory links, Email
Search Function		Across populations, practices, Export functions
Patient access/Control		Access to information through web portals, etc. Attribution
**Processes–care and review**		
	Quality Markers	Data quality, information quality, system accreditation
	Billing/Pay for Performance	Routine data use, parallel billing system
	Supports population level data outputs	Small area, sentinel networks, epidemiology
	Processes that impact on outcomes (demonstrated within the system)	Critical incidents / near misses / confidential reporting; surrogate markers of quality and outcomes/Clinical audit; true outcome measures

**Software packages reviewed.**

Medical Director 2 (Health Communication Network, Sydney, NSW)

Best Practice (Best Practice Software, Bundaberg, Queensland)

Genie (Genie solutions, Brisbane, Queensland)

Medtech32 (Medtech Global, Melbourne, Victoria)

Plexus (iSoft, Sydney, NSW)

Profile (Intrahealth Systems, Sydney, NSW)

Promed (Promedicus Systems, Melbourne, Victoria)

**Table 2:** CMR and CG maturity model: moving through passive, interactive, and autonomous modes.

Level 1	Level 2		Level 3	Level 4
Simple			Complex	Adaptive systems
External adverse event reporting (no use of system)		Reporting involving information from CMR	Reporting using the CMR as vehicle	Interactive reporting where CMR sends and receives information, informing user of the risks
Simple prescribing		Prescribing with limited functions (interaction checking)	“Intelligent” prescribing where CMR uses local information such as guidelines to inform prescribing decisions	“Autonomous” prescribing where system integrates internal and external information to determine optimal management
Simple audit feedback loops		Audit data compared with external data to assess performance	Audit data pooled and used to develop local benchmarks as well as population health activities	Real-time data aggregation and assessment to allow ‘just in time’ monitoring of population, during pandemics, for example
Largely External to CMR		Integrated in CMR	CMR linked to other information sources	Integrated into health system
Distributed database Isolated Linkage Integration				Interoperable data

## Results

### Context

#### Organizational

In Australia primary care is delivered via general practice through around 7000 discrete practices. Practices in Australia have a variety of ownership structures including corporate owners, partners, associates, and sessional general practitioners (GPs). Some CMRs enabled varying degrees of control according to status within the practice-owner, employee, etc. Patients are free to visit any GP of their choice, and GPs act as gatekeepers to secondary care. Mobility of patients between practices means that they lack the stable population denominator of registration based systems such as those found in the UK or Netherlands. Funding is largely fee-for-service underpinned by a national insurance scheme, but there are many accessory payments [27] and other programs [28]. The CMR systems also allowed for different role-based access for nurses and receptionists. Standards for clinical governance have been introduced by the Royal Australian College of General Practitioners [29].

#### The Individual Clinician

The GPs in Australia are trained in the Australian General Practice Training Program. The curriculum for training [29] includes a specific section on eHealth focussed on the practical use of computers, but not their application as a tool for clinical governance. A total of 98% of GPs have a computer on their desk, which they use them for clinical purposes [30]. Most GPs use their CMR for recall, maintenance of immunization registers, monitoring of population health, making clinical notes, and/or recording diagnoses using a clinical coding system [30]. There are some 22+ clinical packages in the market. Over 40% of GPs are involved in some sort of audit or quality assurance cycle associated with using their computer data, usually mediated by the local Division of General Practice [31]. These activities require good data and appropriate extraction techniques.

#### Technology

Although doctors use many sources of information in the consultation [32], it is the clinical packages that can have the largest impact on the clinical outcomes. In general practice, the government has encouraged good data recording through its Practice Incentive Program. Practices have received payments for recording allergies and the creation of summary data in their CMRs.

We identified four technological issues that compromise clinical governance activities:

- Different (and local) coding systems make national and international comparison of quality more challenging.
- The absence of standards meant CMR vendors can choose to develop and implement their own messaging ‘standard’ for use between variants of their program including use of varying flavors of Health level-7 (HL7), with much less scope for quality control and minimizing the risk of inbuilt errors.
- Patient access to the record was absent. Such facilities are not part of the Australian landscape yet.
- Backup facilities were not inbuilt functions of the software, but were integrated into general system backups according to accepted guidelines.

#### Clinical Task

The individual clinician had little influence on the software processes. In comparison with paper records, we felt the CMR disempowered the clinicians–in effect ceding many areas of control to the organization or the technology. Customization options were minimal. Some programs did not allow individual doctors to change their passwords without going through an administrator. Access controls for all staff were either set by the program or customisable by a designated administrator.

A significant amount of data required to perform key clinical tasks is now provided by third parties, who have to be trusted themselves to have proper governance systems. The responsibility, governance, and overall control of these information sources sit outside of the CMR. For example, drug information was derived from either government sources or from the industry. Until 2009, the most popular general practice software incorporated screen drug advertising. An audit of these advertisements found that 95% were non-compliant with the Medicines Australia Code of Conduct, though there was a little evidence that this impacted upon prescribing practice [33,34]. Most programs sourced travel medicine advice from a variety of industry sources. Immunization schedule data was the one area that used a common, validated source (the federal government).

There are significant gaps and variability between Australian CMR systems in their drug interaction checking [35], though these issues are international [36]. While there are standards about CMR functionality they largely fail to include how applications should perform in clinical environments [37] especially as the CMR becomes more ‘active’ in the patient space [38].

Some areas were easy to ascribe to an actor, but others were quite complex. Drug Interactions, for instance, required taking an externally provided database, integrating it into the program, and then allowing GPs to potentially customize the level of alert setting, and then integrate all of that into the consultation. Others such as practice audit required a reliable software process that was then dependent on a practice system to make best use of the information.

**Table 3:** Contextual elements that support and limit clinical governance using computerized medical records.

Context	Reviewed Elements
Organizational	Accessible by different cadres of practice staff^a^
	Accreditation standards includes clinical governance^a^
	Patients are not enrolled, and can be very mobile^b^
Individual clinician	Clinicians receive training in operating computers^a^
	Nearly half of Australian GPs are involved in quality audits^a^
Clinical task	Individual clinicians have little autonomy over the software system, and must respond to its settings^b^
Technology	Variety in coding systems^b^
	Lack of standardization^b^
	No patient access^b^
	No back-up systems for CMR itself^b^

^a^Contextual elements that support clinical governance using computerized medical records.

^b^Contextual elements that limit clinical governance using computerized medical records.

### Mechanism: The CMR

#### Mechanism: Structures

All systems generated a unique identifier for each patient, and all recorded the Medicare number (a non-unique number used for the federal insurance scheme). All CMR systems utilized a graphical user interface and all had standard clinical archetypes such as history, examination, past history, and social history. All were able to provide a summary view although differences in those views were apparent [39]. All were able to code diagnosis and problem list data, although four different coding systems were used: International Classification of Primary Care, International Classification of Disease version 10 (ICD-10), Pyefinch, and Doctor Command Language. One system that used the ICD-10 classification incorporated the ICD-10 procedure code; thus, the system included more extensive classification on complications of cataract procedures than it did on hypertension. The system required so much clicking to turn off the classification system that doctors reported bypassing the classification system altogether.

None used the Systematised Nomenclature of Medicine–Clinical Terms, the official Australian standard and none required data to be entered in a coded fashion, and two of the coding systems are specific to that brand of software. All the CMR systems allowed attribution of data according to login or according to source. Some incoming data (such as specialist letters) required manual attribution, while for data such as pathology, the attribution was automatic.

Every CMR system was able to accept pathology and radiology as atomized data (either HL7 or Pathology Interchange Format). All programs allowed linking of requests with received reports. Four packages allowed both generation of electronic documents and receipt. All used proprietary systems to do this, with little ability to work cross platform.

The CMR systems (in keeping with the genesis of software systems as electronic prescribing packages) had comprehensive drug databases. Most used the database from MIMS Australia, otherwise using information from a variety of sources. Data regarding Australia’s Pharmaceutical Benefits Scheme (PBS), which detailed government subsidies for most drugs, was sourced from the PBS itself. All had the ability to generate drug interactions, although users were able to set the level of drug interaction alerts and in several systems turn them off altogether. Use and availability of drug calculators (weight/dose calculators or warfarin calculators) was extremely variable. All packages had a variety of other external information sources available from within the system.

All CMR systems had immunization information; many had travel information, and one had an extensive library of text based health information resources within the program.

All programs have search functions built into the system. Most have some inbuilt searches (patients over 65 years, eligible patients without a cervical smear in the last five years) that relate to funding initiatives or chronic disease management. The ability to do other searches was quite variable and often required significant computer/database knowledge

### Mechanism: Processes of Care and Review

Only four of the CMR systems were able to participate in regional data quality activities. These activities revolve around the Australian Primary Care Collaborative program, The Practice Health Atlas and the PEN Clinical Audit tool [31]. All these activities require the use of an external tool to interrogate the program’s database and generate pooled data. One other package had its own tool to perform similar functions. All programs were able to generate pay-for-performance lists, according to the particular funding initiative.

### Mechanism: Processes That May Impact on Outcome

No system had inbuilt data quality checks (prescribing insulin without a diagnosis of diabetes for example). One system had its own ‘in-house’ sentinel/research network ability; no other program had such a designated function.

**Table 4:** Mechanisms that support and limit clinical governance from computerized medical records.

Mechanism	Reviewed Elements
Structures	External resources (eg, MIMS) included^a^
	Alert to drug interactions^a^
	Accept pathology and radiology results as atomised data^a^
	Limited search facilities^b^
	Variable drug dose calculators^b^
	No standardized coding system^b^
Processes of care and review	Can generate pay for performance^a^
	Half allow data extraction to participate in audits^b^
Processes related to outcomes	No inbuilt data checks for quality^b^
	Only one allows in-house sentinel data search facility^b^

^a^Mechanisms that support clinical governance using computerized medical records.

^b^Mechanisms that limit clinical governance using computerized medical records.

### Outcomes

#### Overview

When context (Table 3) and mechanism (Table 4) were explored together, we found that the contextual limitations associated with the technology landscape and clinician autonomy over the CMR compounded the limitations identified in the analysis of mechanisms, associated particularly with processes. The result is that these systems have limited demonstrable outcomes in relation to clinical governance.

#### Demonstrated Outcomes for the Patient or for the Health Care Provider

Most medical records are computerized and widely used for clinical governance activities, but these approaches are fragmented [16]. None of the packages dealt effectively with health outcomes, in the sense that they were able to adequately demonstrate improved care from within their own processes. Assessing health outcomes required an interpretive process by accessing and comparing external data. The tool asked for ‘surrogate markers of quality’ and ‘outcome measures’, neither of which was particularly well or sufficiently defined to be assessed. However, in the future, these features will become of prime importance.

#### Outcomes at Population Level

None of the CMRs was able to deal directly with these issues. However, the ability of the systems to provide data to inform activities at this level is increasingly crucial for health system management if we want to be able to explore what population interventions might have impact. Medicare locals, the regional support bodies for primary care services, are able to use the data for informing practice at a local level [40], but the ability for this data source to influence national activities is currently poor.

### Maturity Framework

At the structural level, Australian CMRs are well developed but there is scope for further progress against our maturity framework. Lacking are open standards, as yet no implementation of a standard coding system, and probably too many vendors in a relatively small market.

Australian primary care is therefore largely at level 2, with some systems only supporting level 1 and with some systems offering level 3 models. There was no evidence of level 4 systems. Some CMR systems had features that from the international perspective must be a developmental blind alley. The local coding systems are one of these; it is unlikely to ever become part of an interoperable health system.

## Discussion

### Principal Findings

In the Australian context, at practice and locality level the CMR works well, and is being used to facilitate clinical governance activities. Nearly all practices have systems with search functionality that enable participation in clinical audit.

However, while practices and localities are widely engaged in clinical governance processes, these are usually being done in an uncritical way. In particular, there is little attention given to data quality, or the obligation to code clinical conditions in standardized extractable fashion.

The record structures are often proprietary and there is a dearth of open architectural models, with many mission critical functions happening within a black box.

### Implications of the Findings

Benchmarking standards at a national or international level will be challenging if poor data quality and the disparate nature of record systems and system architecture remain unaddressed. Although not a registration based system, denominators such as those who attended in the last year can be used to make comparisons between practices and systems.

It is not possible to have lossless conversion of data held in one coding system to another, and the use of idiosyncratic coding systems increases the risk of data loss. While statistical techniques, in particular multiple imputation [41], can be used to compensate for missing data, this is never the same as having complete data. Black box data extraction processes and audit systems tend to foster uncertainty about the validity of findings.

Disease registers are much more challenging to set up when there is incomplete coded data, and patients with a condition not on a disease register are not going to benefit from computerized prompts or recall. Their standard of care may also be lower. This data quality and use issue will become a major problem as more information is shared.

### Comparison With the Literature

The complexity of the clinician-patient-computer interaction, touched on in the introduction, is reflected elsewhere in the literature. Patient-centered care [38,42] and relationship-centered care [43] have taken hold and been shown to affect the outcomes. Computerization is changing the balance of power in the interaction [44].

There is no requirement for CMR systems to provide any specific functionality whatsoever, no set of criteria over information use, and no standards over usability or even formally recommended testing protocols [37]. The ‘Swiss cheese’ model of error [45] highlights how gaps in complex systems can result in errors, which in turn can raise patient safety issues. Drug interaction checking is an example of this, with interaction resources needing to be integrated into the prescribing package and then used by the clinician. While the UK National Program for IT has been much criticized, the one area that appears to have stood the test of time has been the rigid implementation of a drug dictionary and messaging standards [46].

Patient access to their records has become the norm elsewhere [47] and increased openness may help ensure good governance. Australia has aspirations to provide patient access through the National “Personally Controlled Electronic Health Record” program. Online access is no panacea; however, uptake of access to very different models of online summaries of care has been poor uptake in both England and France [48].

A comparison with the UK system of CMR driven pay-for-performance suggests that there may be quality gaps that computer mediated incentives might help close [49,50]. Additionally, the UK Primary Care Information Service (PRIMIS) has promoted data quality through a wide range of initiatives. The PRIMIS approach has been one of facilitation and feedback rather than financial incentives. These have been clinically focussed and included looking at disparities in data quality heart disease and improving patient safety [51,52]. However, more recently, the English NHS has gone through a game changing transformation with extraction of data on a National Scale through a system called the General Practice Extraction Service This gives the potential to extract data to measure quality and clinical governance on a national scale. The GPES system has its own Independent Advisory Group (SdeL is the Royal College of General Practitioners representative) to: *“Consider the risks and benefits in order to assess whether the extraction is in their view appropriate and in the public interest.”*

### Limitations of the Method

The evaluation took place at a point in time in 2009, and each package has gone through several upgrades over that time. As such, this analysis is not meant as a detailed critique of the packages with recommendations. It is quite possible that many of the comments here may no longer apply to a specific package. Moreover, it is the first discussion of the increasing influences on clinical governance by CMRs, with a framework that can be applied serially or in different contexts. What is lacking in planning and development is a consistent approach to thinking about CMRs and clinical governance, and what systemic controls should be there.

We might have explored the extent to which the standardization of record formats might have aided comparison and measurement of quality. The Royal College of Physicians, UK, has been very active in trying to standardize records reporting handovers, including admission and discharge [53]; it is likely that such a process would facilitate the implementation of clinical governance process. Although we make reference to the Open EHR initiative in our method we have not fully described its potential impact on standardization, and therefore toward being able to contribute to governance by facilitating the measurement and compare clinical standards on different systems. The two elements of Open EHR we believe that contributes most are its clinical models program, which enables researchers and practitioners to build sharable archetypes of clinical concepts [54]; and the specification program that defines data, services, and application program interfaces and offers the allure of quality certification of systems [55].

There are also other models we might have used for example: Yousuf et al have proposed an adoption model that includes: user attitude and skills base together with good leadership, IT-friendly environment, and good communication [56]. Lau et al have identified factors that influence adoption, and that it includes people, organization, implementation, and the macro environment [57].

These models share some similarity in that they both identify socio-technical aspects of implementation. Had we used either of these models, our subheadings might have been different but our findings are unlikely to have changed. Our selection driven by the wish to emphasize the interaction between organization (which included governance), the individual clinician, the clinical task (which should be of quality) and the technology; and not predefined success factors or progressive levels that should be reached.

### Call for Further Research

The observations in this study have not been tested in a controlled trial and are retrospective in nature. Although we have approached this study from a neutral position of identifying factors that helped and hindered there may be bias. One author (CMP) is very familiar with many of the brands of Australian CMR and may have been susceptible to *familiarity bias* [58], and pointed out issues he was previously aware of. However, SdeL does not share this bias, instead having his experience framed in other countries’ CMR systems. Our assertion is that the CMR is as an instrument of and for clinical governance. At the very least, the CMR provides the tools to enable clinical audit and retrospective analysis of data. At its best the CMR can flag, recall, remind to monitor, and provide information support, and taking an ever more active role in the consultation. The current use of CMRs in Australia supports clinical governance at the individual patient, practice and possibly locality level; but provides no insights at the national level. Where the CMR does not facilitate clinical audit, individual practitioners are blocked from raising quality standards. We need to further test this hypothesis in prospective trials.

### Conclusions

We have developed a framework for evaluating how CMR systems support clinical governance in a particular context; and whether the CMR has helped to achieve those goals. By applying the tool to several different brands of Australian CMRs, we have highlighted the issues that exist today, but importantly shown a graded way forward using a simple model and maturity framework that we hope can be readily followed by clinician users of these systems.

The limitations of the process relate to the heterogeneity of the data and their sources, the continuing change over time, but above preeminent is the lack of implementation of standards. While CMR implementation in Australia has enabled better clinical governance improving systems technical capability and rigorous standardization is needed to enable more comprehensive assessment of quality and outcomes for patients.

## Multimedia Appendix 1

Software assessment schema.

## Abbreviations

CMR: computerized medical records
EHR: electronic health record
GP: general practitioners
HL-7: Health level-7
ICD-10: International Classification of Disease version 10
NHS: National Health Service
PBS: Pharmaceutical Benefits Scheme
