# Supplementary material for: The Computerized Medical Record as a Tool for Clinical Governance in Australian Primary Care
Source: Interact J Med Res. 2013 Aug 12;2(2):e26. doi: 10.2196/ijmr.2700 (PMC3744386; doi:10.2196/ijmr.2700)
Supplement: Supplementary file 1 [file ijmr_v2i2e26_app1.pdf]

**STRUCTURES**		**Individual clinician**<br>*Their knowledge of, skill in operation & attitudes towards EPR use for governance*	**Clinical task**<br>*Usually the 1 to 1 clinical consultation – may involve implementing guidelines*	**Technology**<br>*With its primary & secondary roles – compare available technology with list Secondary roles may include pay-for-performance*	**Organisation**<br>*Practice, Locality, Region, and National drivers/priorities*
**System architecture**					
	Interface (GUI, CHUI etc.)				
	Clinical archetypes				
	Database (closed, restricted, open API – Local/Hosted)				
	Coding system				
	Recalls (set/free)				
	Shared record capability				
	Audit trail				
	Attribution				
	Security, back-up, system recovery, service support				
**Information support**					
	Information retrieval system				
	Drug information database				
	Guidelines				
	Clinical information				
	Types				
	Source (internal/external)				
	Clinical decision support				
	Drug/drug interactions				
	Drug/condition interactions				
	Drug adverse reaction				
	Warfarin dosage				
	Immunisation				
	Clinical				
**System linkage (and if to national / international standards)**					
	Patients				
	Registration database				
	Unique ID				
	Laboratory				
	Imaging				
	Clinic				
	In-patients				
	Email & Web				
	Referral				
	Others				

**STRUCTURES**		**Individual clinician** *Their knowledge of, skill in operation & attitudes towards EPR use for governance*	**Clinical task** *Usually the 1 to 1 clinical consultation – may involve implementing guidelines*	**Technology** *With its primary & secondary roles – compare available technology with list Secondary roles may include pay-for-performance*	**Organisation** *Practice, Locality, Region, and National drivers/priorities*
**Search function**					
	Population				
	Practice				
	Linked data				
	Export functionality – and common interlink formats				
	Others				
**Patient access / control**					
	Access to record (Read only)				
	Add to record (Write)				
	Ownership of record (Control access)				
	Access to pooled quality data				
	Others				
**PROCESSES**					
**Quality markers**					
	Denominator quality				
	Data quality				
	Clinical quality – participation in audit – practice / locality / national				
	Fitness for practice – Appraisal / validation				
	Complaints				
	Accreditation (of the system)				
	Training (of the users and their supervisors – e.g. Caldicott guardian)				
**Billing pay for Performance**					
	Routine data used for pay for performance				
	Local				
	National				
	Separate billing process data				
	Perverse incentives				
**Epidemiology /Health needs assessment**					
	Epidemiology / sentinel networks				
	Health service management / needs assessment				
	Safety and surveillance				
**OUTCOMES**					
**1)**	**Surrogate markers of quality**				
**2)**	**Critical Incident Reporting**				
**3)**	**Outcome markers**				
